# A Fluorescence Resonance Energy Transfer Probe Based on DNA-Modified Upconversion and Gold Nanoparticles for Detection of Lead Ions

**DOI:** 10.3389/fchem.2020.00238

**Published:** 2020-04-21

**Authors:** Yue Wang, Menghua Lv, Zehan Chen, Zilong Deng, Ningtao Liu, Jianwei Fan, Weixian Zhang

**Affiliations:** ^1^State Key Laboratory of Pollution Control and Resources Reuse, College of Environment Science and Engineering, Tongji University, Shanghai, China; ^2^Department of Neurosurgery, Shanghai Tongji Hospital Affiliated to Tongji University, Shanghai, China; ^3^Shanghai Institute of Pollution Control and Ecological Security, Shanghai, China

**Keywords:** upconversion nanoparticles, gold nanoparticles, fluorescence resonance energy transfer, DNA, lead ions

## Abstract

We report a new sensor for the specific detection of lead ions (Pb^2+^) in contaminated water based on fluorescence resonance energy transfer (FRET) between upconversion nanoparticles (UCNPs) as donors and gold nanoparticles (Au NPs) as receptors. The UCNPs modified with Pb^2+^ aptamers could bind to Au NPs, which were functionalized with complementary DNA through hybridization. The green fluorescence of UCNPs was quenched to a maximum rate of 80% due to the close proximity between the energy donor and the acceptor. In the presence of Pb^2+^, the FRET process was broken because Pb^2+^ induced the formation of G-quadruplexes from aptamers, resulting in unwound DNA duplexes and separated acceptors from donors. The fluorescence of UCNPs was restored, and the relative intensity had a significant linear correlation with Pb^2+^ concentration from 0 to 50 nM. The sensor had a detection limit as low as 4.1 nM in a buffer solution. More importantly, the sensor exhibited specific detection of Pb^2+^ in complex metal ions, demonstrating high selectivity in practical application. The developed FRET prober may open up a new insight into the specific detection of environmental pollution.

## Introduction

Lead ion (Pb^2+^), one of the most serious metallic toxicants, can damage cardiovascular, reproductive, neurological, and developmental systems of the human body at low concentrations in the blood (Yoosaf et al., 2007; Zhou et al., 2011; Kim et al., 2012; Li et al., 2013). Although traditional methods including inductively coupled plasma mass spectrometry (Xia et al., 2008; Gao et al., 2009), atomic absorption spectroscopy (Bravo-Sanchez et al., 2001), and high-performance liquid chromatography (Yang et al., 2003) are highly sensitive and accurate, complicated pretreatment, and implementation limit their applicability for on-site rapid detection. Therefore, it is of important significance to develop analytical strategies with facile and straightforward features.

The detection assay composed of fluorescence and DNA molecules has attracted more and more attention mainly due to sensitive fluorescence signal, stable DNA molecules, and highly specific binding ability between specific sequences and target detectors (e.g., protein, ions, virus, and nucleic acid aptamers) (Hamaguchi et al., 2001; Pavlov et al., 2004; Xiao et al., 2005; Chang et al., 2010; Saha et al., 2012). Zhou and co-workers labeled 6-carboxyfluorescein on G-rich DNA strands and monitored the reduction of fluorescence for Pb^2+^ detection (Zhan et al., 2013). Shi and co-workers developed a new strategy based on DNA-templated silver nanoclusters with elevated fluorescence for L-histidine detection (Zheng et al., 2015). However, reported substances or quenchers in the actual environment might weaken the fluorescence and lead to false-positive results. Therefore, the “turn-off–on” detection system based on fluorescence resonance energy transfer (FRET) is introduced to avoid external interferences.

Upconversion nanoparticles (UCNPs) have rapidly emerged owing to their unique luminescent properties (Wang et al., 2005; Mader et al., 2010; Haase and Schaefer, 2011; Chen et al., 2014). Compared to traditional fluorescent markers, the UCNPs presented low auto-fluorescence, narrow emission width, no flicker, and strong light stability, leading to a wide application in biological and environmental monitoring and sensing (Chen and Zhao, 2012; Chen et al., 2013, 2014; Dacosta et al., 2014). Therefore, upconversion luminescence, as sensing signal under excitation of the near-infrared ray (NIR) light, effectively diminished the background noise in a complicated detection system (Wu et al., 2014). Meanwhile, gold nanoparticles (Au NPs) are superior fluorescence quenching agents due to the large extinction coefficient and a wide absorption band in the UV–visible region (Peng et al., 2011; Lin et al., 2013; Liu et al., 2013). The FRET systems were established based on upconversion nanoparticles and gold nanoparticles for the detection of avidin and Cr^3+^ (Wang et al., 2005; Liu et al., 2013).

In this paper, NaYF_4_: Yb, Er @NaYF_4_ UCNPs as energy donors and Au NPs as energy receptors are employed as FRET system for the sensitive detection of Pb^2+^. The donors and receptors are paired by two complementary DNA strands with good quenching ability for UCNPs. Single-stranded DNA for modifying UCNPs is rich in G base, which can fold to G-quadruplex structure in the presence of Pb^2+^. DNA duplex is then disrupted and the FRET system between UCNPs and Au NPs is cleaved, resulting in the restoration of fluorescence. The concentration of Pb^2+^ can be detected by monitoring the fluorescence recovery.

## Materials and Methods

### Materials

Anhydrous yttrium trichloride (YCl_3_, 99.99%), anhydrous ytterbium trichloride (YbCl_3_, 99.9%), anhydrous erbium chloride (ErCl_3_, 99.99%), 1-octadecene (ODE, 90%), oleic acid (OA, 90%), sodium hydroxide (NaOH, 96%), and ammonium fluoride (NH_4_F, 96%) were purchased from Sigma-Aldrich. Trihydroxy methyl aminomethane (Tris), HCl, NaCl, KCl, CaCl_2_, MgCl_2_, CuCl_2_, ZnCl_2_, and FeCl_3_ were obtained from Sinopharm. All chemicals were used directly without any further purification. Deionized water was purified by a Milli-Q system (Millipore, Bedford, MA, USA). The lead standard solution (1,000 mg/L) was purchased from Aladdin Industrial Inc. All nucleic acid molecules were prepared by Bioengineering Co., Ltd. (Shanghai). The sequences were as follows:

DNA1: 5′>AAGGGT GGGT GGGT <3′

DNA2: 5′>AAAAA AAAAA AAAAA AAAAA TTTTT CACCC TCCC AC <3′

### Synthesis of NaYF_4_: Yb, Er

The NaYF_4_: 18% Yb, 2% Er UCNPs were prepared according to the previous report (Li and Zhang, 2008). Typically, YCl_3_ (0.80 mmol), YbCl_3_ (0.18 mmol), ErCl_3_ (0.02 mmol), OA (6.0 ml), and ODE (15 ml) were mixed and heated to 140°C under vacuum for 1 h before cooling down to room temperature. Thereafter, NH_4_F (4.0 mmol) and NaOH (2.5 mmol) in methanol (10 ml) was added to the resulting solution and stirred for 30 min. The mixture was then transferred to a vacuum oven at 70°C for 30 min and heated at 300°C under argon flow for 1 h. NaYF_4_: 18% Yb, 2% Er cores were obtained as final product. As-prepared nanoparticles were washed with ethanol for several times and dispersed in 10 ml of cyclohexane.

### Synthesis of NaYF_4_: Yb, Er @NaYF_4_ UCNPs

To prepare NaYF_4_: Yb, Er @NaYF_4_ UCNPs, YCl_3_ (0.25 mmol), OA (6.0 ml), and ODE (15 ml) were mixed and transferred to a vacuum oven at 140°C for 1 h. The solution was added with NaYF_4_: Yb, Er initial core solution (5 ml) after cooling down to room temperature and heated at 70°C in a vacuum oven for 30 min to remove cyclohexane. Subsequently, the obtained mixture was further maintained at 280°C under argon atmosphere for 1 h. The NaYF_4_: Yb, Er @NaYF_4_ UCNPs were washed before dispersing in cyclohexane.

### Surface Modification of NaYF_4_: Yb, Er @NaYF_4_ UCNPs

Ligand-free UCNPs were synthesized ahead of DNA modification following the method reported by Bogdan et al. (2011). The oleic acid-capped UCNPs in cyclohexane were centrifuged by adding ethanol as precipitant. Then, 100 mg of UCNPs was mixed with 10 ml water, and the pH of the solution was adjusted to 4 with 0.1 M hydrochloric acid solution. The solution was extracted three times with diethyl ether after stirring for 2 h, and the nanoparticles obtained were transferred to the aqueous layer and precipitated with acetone. Afterward, the oleic acid-ligand layer was removed and the ligand-free UCNPs were dispersed in water (5 ml).

DNA1 (200 nmol) was added to the solution of ligand-free UCNPs (20 μmol Ln^3+^). Following stirring overnight at room temperature, the mixture was centrifuged to remove excessive DNA1. The resulting DNA1-modified UCNPs were dispersed in Tris-HCl buffer solution (20 mM, 1 mM MgCl_2_, 2 mM KCl, and 100 mM NaCl, pH 7.4) and stored at 4°C.

### Preparation of DNA2-Modified Au NPs

Au NPs were prepared based on the previously reported method (Chen et al., 2012; Pei et al., 2012). The boiling HAuCl_4_ (HAuCl_4_·4H_2_O, 99.99%) solution was added with trisodium citrate solution (1%) with 20 min of stirring and then cooled down to room temperature to obtain Au NPs.

DNA2 was added to citrate-stabilized Au NPs with a stoichiometric ratio of 1:50, and a tiny amount of sodium citrate-hydrochloric acid buffer solution (500 mM, pH 3.0) was then rapidly added to the said mixture to make a final concentration of 10 mM. The solution was centrifuged for several times to remove excessive DNA2. DNA2-modified Au NPs were yielded and dispersed in Tris-HCl buffer solution (20 mM, pH 7.4).

### Detection of Pb^2+^

The concentration of DNA1-modified UCNPs was immobilized, and DNA2-modified AuNPs with varied concentrations were added to the system. The mixture was incubated for 90 min at 37°C. The optimal concentration of AuNPs-DNA2 was determined by the fluorescence quenching efficiency of UCNPs-DNA1.

The Pb^2+^ standard solution with different concentrations was added to a mixed solution including UCNPs-DNA1 and AuNPs-DNA2, which had been incubated for 90 min. The said solution was further incubated for 30 min to measure its fluorescence spectra.

### Characterization

Transmission electron microscopy (TEM) images were taken with a JEM-2100F microscope (200 kV, with a Gatan imaging system). The upconversion fluorescence spectra were performed on a Hitachi F4500 fluorescence spectrometer (xenon lamp excitation source with a 980-nm laser). The UV–vis absorption spectra were characterized by a Lambda 750S UV/Vis/NIR spectrometer.

## Results and Discussion

### Principle of UCNPs-AuNPs FRET Sensor for the Detection of Pb^2+^

**Figure 1:**
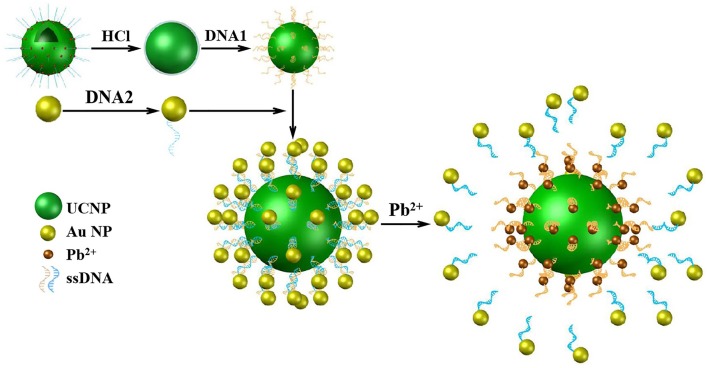
The schematic illustration of sensing assay constructed by UCNPs and Au NPs for Pb^2+^ detection.

The sensor for Pb^2+^ detection was based on FRET from modified UCNPs to Au NPs by an aptamer matching its complement (Figure 1). Firstly, UCNPs were treated with hydrochloric acid to remove the hydrophobic surface ligands and modified with the aptamers (DNA1). Au NPs were functionalized with the complementary DNA (DNA2). Secondly, the FRET system was established with UCNPs as donors and Au NPS as receptors. The distance between UCNPs and Au NPs was shortened to <10 nm because of complementary DNA hybridization, leading to quenched fluorescence of UCNPs. Thirdly, the aptamers preferred to bind with metal ions and turned into intermolecular G-quadruplexes in the presence of Pb^2+^. Therefore, the DNA duplexes were unwound and upconversion fluorescence was restored to determine the concentration of Pb^2+^.

### Characterization of DNA-Modified Nanoparticles

**Figure 2:**
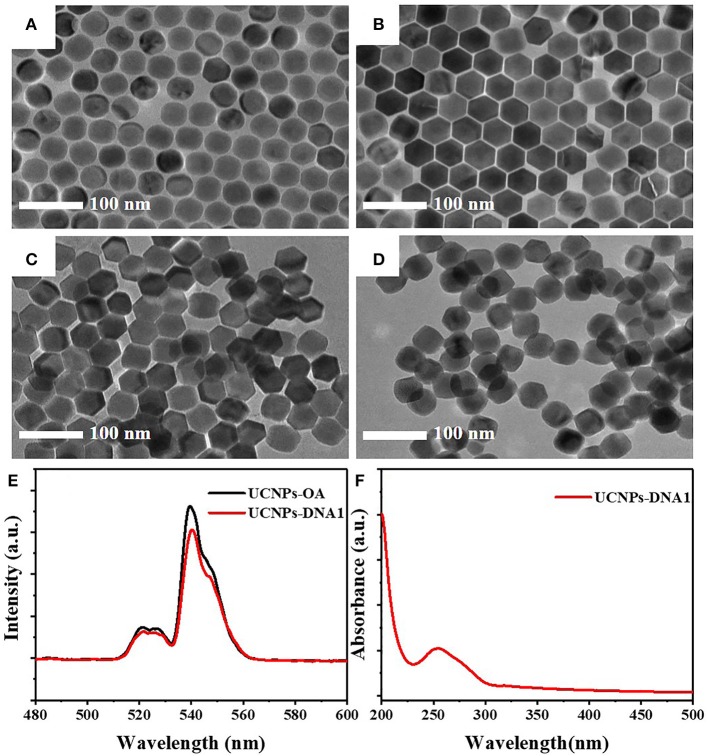
TEM images of **(A)** NaYF_4_: 18% Yb and 2% Er, **(B)** NaYF_4_: 18% Yb and 2% Er @NaYF_4_, **(C)** ligand-free UCNPs, and **(D)** DNA1-modified UCNPs (UCNPs-DNA1). **(E)** The upconversion fluorescence spectra of UCNPs with OA surface ligand (UCNPs-OA) and DNA aptamers (UCNPs-DNA1) under excitation of 980 nm. **(F)** The UV–vis absorption spectra of UCNPs-DNA1.

The NaYF_4_: 18% Yb, 2% Er with a diameter of 45 nm was fabricated by the solvent–thermal method in Figure 2A (Li et al., 2014) and further coated with the NaYF_4_ passivation shell for the formation of NaYF_4_: 18% Yb, 2% Er @NaYF_4_ core/shell-structured UCNPs with a diameter of about 50 nm (Figure 2B). The OA ligands capped on UCNPs were treated with hydrochloric acid to form the water-soluble ligand-free UCNPs (Figure 2C). The TEM image shows that the obtained water-soluble ligand-free UCNPs remained uniform and monodispersed. Upon DNA1 modification, the UCNPs retained good dispersion with negligible morphological and size alteration (Figure 2D). The fluorescence of UCNPs was slightly quenched by water after being modified with DNA1 (Figure 2E). A strong absorption peak at 260 nm from the DNA was clearly observed in the spectrum of the DNA1-modified UCNPs, confirming that the UCNPs were successfully modified with DNA-1 (Figure 2F).

**Figure 3:**
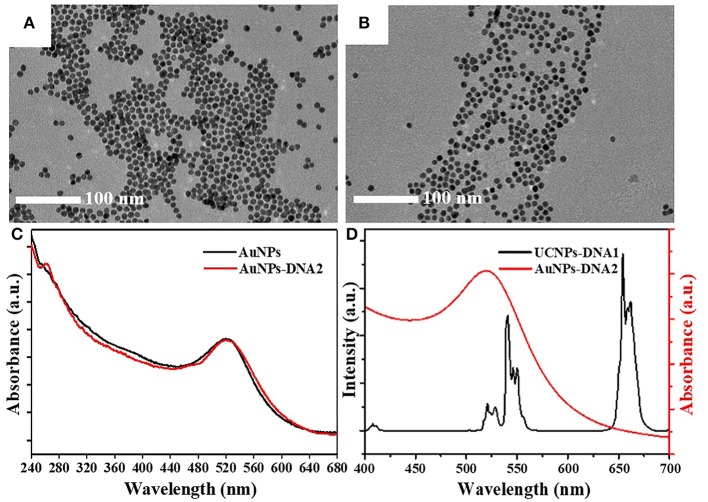
TEM images of **(A)** Au NPs and **(B)** DNA2-modified Au NPs (Au NPs-DNA2). **(C)** The UV–vis absorption spectra of Au NPs and Au NPs-DNA2. **(D)** The upconversion fluorescence spectra of UCNPs-DNA1 and the UV–vis absorption spectra of Au NPs-DNA2.

Au NPs with a diameter of 10 nm (Figure 3A) were modified with DNA2 molecules, which were partially complementary with DNA1. The DNA2-modified Au NPs remained of good dispersion (Figure 3B). The significant DNA absorption peak was observed at 260 nm, while the maximum absorption peak of Au NPs at 520 nm presented no significant variation (Figure 3C). A spectral overlap was illustrated in the range of 510–570 nm between UCNPs and Au NPs (Figure 3D).

### Optimization of FRET System

**Figure 4:**
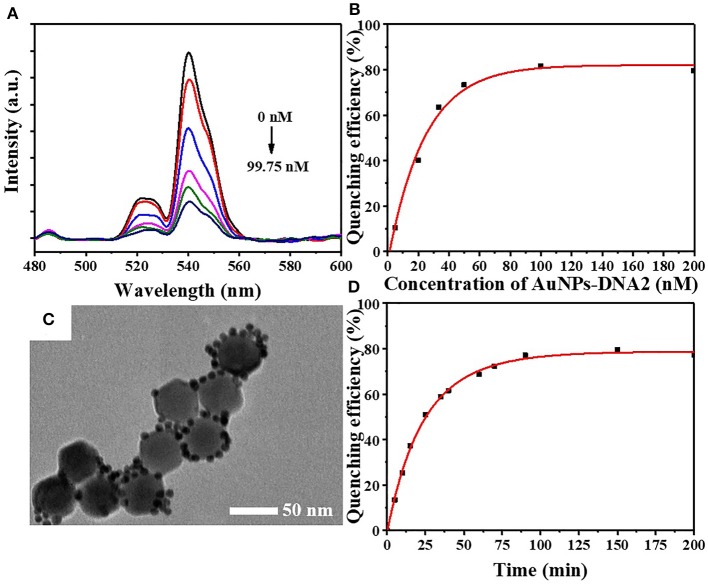
**(A)** The upconversion fluorescence spectra and **(B)** quenching efficiency of UCNPs-DNA1 after incubation at various concentrations of AuNPs-DNA2 in FRET system. **(C)** TEM image of FRET array fabricated from UCNPs-DNA1 and AuNPs-DNA2. **(D)** The fluorescence quenching efficiency with increasing incubation time.

The fluorescence intensity of UCNPs gradually decreases along with the elevation of DNA2-modified Au NPs (Figure 4A). The concentration of UCNPs was fixed at 6.56 mg/L accompanied by adjusting the concentration of Au NPs. The quenching efficiency of upconversion fluorescence climbed up to 80% at a concentration of 99.75 nM and remained stable at higher concentrations (Figure 4B). Therefore, the optimal concentration of the DNA2-modified Au NPs is 99.75 nM. In Figure 4C, the UCNPs are surrounded by Au NPs, indicating the construction of the FRET system between UCNPs and Au NPs. Moreover, the fluorescence quenching efficiency reached a plateau (80%) after 90 min of incubation (Figure 4D). Therefore, 90 min of incubation is adopted to achieve a stable fluorescence signal.

### Detection of Pb^2+^

**Figure 5:**
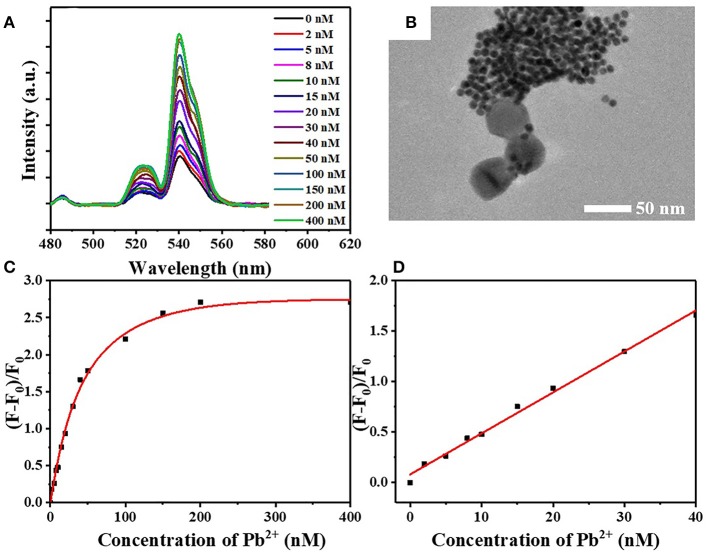
**(A)** The restoration of fluorescence after incubation at different concentrations of Pb^2+^ in FRET system. **(B)** TEM image of FRET array fabricated from UCNPs-DNA1 and AuNPs-DNA2 after Pb^2+^ incorporation. **(C)** The relationship between relative fluorescence intensity (F–F_0_)/F_0_ and Pb^2+^ concentration. **(D)** Linear correlation between relative fluorescence intensity (F–F_0_)/F_0_ and Pb^2+^ concentration in the range of 0–50 nM.

**Figure 6:**
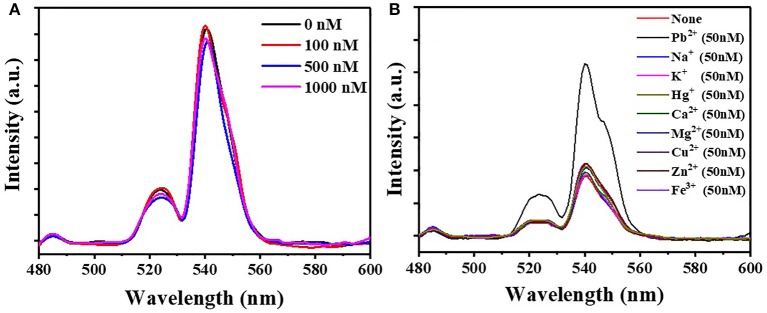
**(A)** The fluorescence intensity of FRET array after incubation at different concentrations of Pb^2+^. **(B)** The fluorescence intensity of detection sensor after the incorporation of Pb^2+^, K^+^, Na^+^, Ca^2+^, Mg^2+^, Hg^2+^, Cu^2+^, Zn^2+^, and Fe^3+^.

The optimal concentrations of UCNPs (6.56 mg/L) and Au NPs (99.75 nM) were chosen for the detection system. After adding the lead standard solution to the system, the DNA aptamers on the surface of the UCNPs were induced to form G-quadruplexes by Pb^2+^, leading to the double-stranded DNA unwinding. Subsequently, the energy transfer system between the UCNP energy donors and Au NP receptors was broken, and the upconversion fluorescence was regained (Figures 5A,B). The fluorescence is restored gradually along with the elevation of concentration (Figure 5C). Linear correlation was demonstrated between the recovery of fluorescence and the concentration of Pb^2+^ in the range of 0 and 50 nM (Figure 5D). Determined to be 3σ, the detection limit of the sensor is 4.1 nM. According to the *Guidelines for Drinking-water Quality* in 2017, the Pb^2+^ concentration was recommended to be no more than 10 ppb or 48 nM (World Health Organization, 2017). Therefore, this study elucidated a great potential of the developed FRET-based assay for *in situ* detection of Pb^2+^ in practice. The effect of Pb^2+^ on upconversion fluorescence was demonstrated by adding different concentrations of heavy metal ions. There is no significant effect on upconversion fluorescence, even if the Pb^2+^concentration was increased up to 1,000 nM (Figure 6A).

### Validation of Selectivity

The selectivity of the sensor for the detection of Pb^2+^ was also tested with different metal ions (Figure 6B, Figure S1). Pb^2+^ ions led to a dramatic fluorescence recovery (177.9%), while metal ions including K^+^, Na^+^, Ca^2+^, Mg^2+^, Hg^2+^, Cu^2+^, Zn^2+^, and Fe^3+^ presented little restoration in the fluorescence spectrum. The result demonstrated the high selectivity of the FRET system for Pb^2+^ detection.

## Conclusions

In summary, a highly sensitive Pb^2+^ detection sensor was constructed based on FRET between UCNPs as donors and Au NPs as receptors. The hydrophobic surface ligands of NaYF_4_: 18% Yb, 2% Er @NaYF_4_ were removed by hydrochloric acid, resulting in enhanced water-dispersible UCNPs which were further modified with DNA1. Au NPs prepared and modified with DNA2, which was partially complementary with DNA1. The FRET assay was fabricated by hybridizing two complementary DNA strands; thus, the green fluorescence of UCNPs was quenched. The specific Pb^2+^ detection was due to the formation of G-quadruplexes derived from the preferred binding between aptamers of UCNPs and Pb^2+^, leading to unwound DNA for the recovery of fluorescence. There was a distinct linear correlation between the relative fluorescence intensity and the concentration of Pb^2+^ from 0 to 50 nM. The developed sensor also presented superior sensitivity (4.1 nM) and selectivity, indicating a promising perspective for Pb^2+^ detection.

## Data Availability Statement

All datasets generated for this study are included in the article/Supplementary Material.

## Author Contributions

YW: formal analysis, investigation. ML: validation. ZC: software. ZD: data analysis, overall planning, and revision of the manuscript. NL: methodology. JF: conceptualization and methodology. WZ: supervision. All authors wrote and reviewed the manuscript.

## Conflict of Interest

The authors declare that the research was conducted in the absence of any commercial or financial relationships that could be construed as a potential conflict of interest.
