# Supplementary material for: A Fluorescence Resonance Energy Transfer Probe Based on DNA-Modified Upconversion and Gold Nanoparticles for Detection of Lead Ions
Source: Front Chem. 2020 Apr 21;8:238. doi: 10.3389/fchem.2020.00238 (PMC7186500; doi:10.3389/fchem.2020.00238)
Supplement: Supplementary file 1 [file Table_1.DOCX]

Supplementary Material


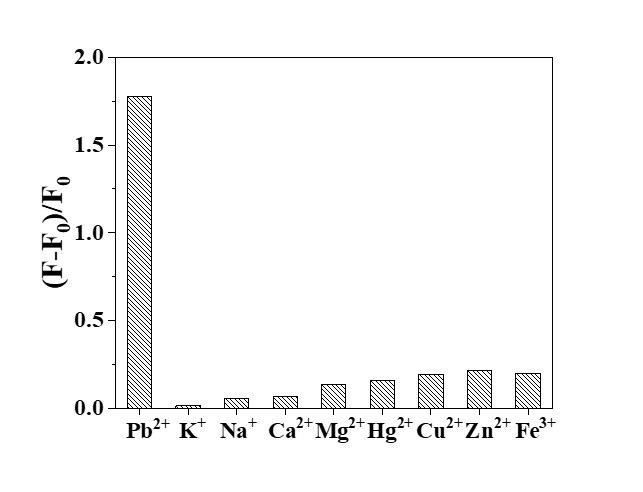


**Figure S1.** Relative fluorescence intensity (F-F_0_)/F_0_ of the FRET system after adding Pb^2+^, K^+^, Na^+^, Ca^2+^, Mg^2+^, Hg^2+^, Cu^2+^, Zn^2+^, Fe^3+^.
